# Variation in the management of cT1 renal cancer by surgical hospital volume: A nationwide study

**DOI:** 10.1002/bco2.229

**Published:** 2023-03-04

**Authors:** H. Yildirim, M. S. Schuurman, C. V. Widdershoven, B. W. Lagerveld, L. van den Brink, A. E. C. Ruiter, H. P. Beerlage, R. J. A. van Moorselaar, N. M. Graafland, A. Bex, K. K. H. Aben, P. J. Zondervan

**Affiliations:** ^1^ Department of Research and Development Netherlands Comprehensive Cancer Organisation Utrecht The Netherlands; ^2^ Cancer Center Amsterdam Amsterdam UMC location University of Amsterdam Amsterdam The Netherlands; ^3^ Department of Urology Amsterdam UMC location University of Amsterdam Amsterdam The Netherlands; ^4^ Department of Urology OLVG Amsterdam The Netherlands; ^5^ Department of Urology Amsterdam UMC location Vrije Universiteit Amsterdam Amsterdam The Netherlands; ^6^ Department of Urology The Netherlands Cancer Institute, Antoni van Leeuwenhoek Hospital Amsterdam The Netherlands; ^7^ The Royal Free London NHS Foundation Trust London UK; ^8^ UCL Division of Surgery and Interventional Science London UK; ^9^ Department for Health Evidence Radboud University Medical Centre Nijmegen The Netherlands

**Keywords:** cT1a renal cancer, hospital volume, renal cell carcinoma, surgical volume, volume standards

## Abstract

**Objectives:**

To analyse variation in clinical management of cT1 renal cell carcinoma (RCC) in the Netherlands related to surgical hospital volume (HV).

**Materials and methods:**

Patients diagnosed with cT1 RCC during 2014–2020 were identified in the Netherlands Cancer Registry. Patient and tumour characteristics were retrieved. Hospitals performing kidney cancer surgery were categorised by annual HV as low (HV < 25), medium (HV = 25–49) and high (HV > 50). Trends over time in nephron‐sparing strategies for cT1a and cT1b were evaluated. Patient, tumour and treatment characteristics of (partial) nephrectomies were compared by HV. Variation in applied treatment was studied by HV.

**Results:**

Between 2014 and 2020, 10 964 patients were diagnosed with cT1 RCC. Over time, a clear increase in nephron‐sparing management was observed. The majority of cT1a underwent a partial nephrectomy (PN), although less PNs were applied over time (from 48% in 2014 to 41% in 2020). Active surveillance (AS) was increasingly applied (from 18% to 32%). For cT1a, 85% received nephron‐sparing management in all HV categories, either with AS, PN or focal therapy (FT). For T1b, radical nephrectomy (RN) remained the most common treatment (from 57% to 50%). Patients in high‐volume hospitals underwent more often PN (35%) for T1b compared with medium HV (28%) and low HV (19%).

**Conclusion:**

HV is related to variation in the management of cT1 RCC in the Netherlands. The EAU guidelines have recommended PN as preferred treatment for cT1 RCC. In most patients with cT1a, nephron‐sparing management was applied in all HV categories, although differences in applied strategy were found and PN was more frequently used in high HV. For T1b, high HV was associated with less appliance of RN, whereas PN was increasingly used. Therefore, closer guideline adherence was found in high‐volume hospitals.

## 1 INTRODUCTION

Renal cell carcinoma (RCC) represents 2%–3% of all cancers diagnosed worldwide.^1^, ^2^ In the Netherlands, the incidence of RCC has risen from approximately 1500 cases per year in 2000 to more than 2600 cases per year in 2020.^3^ Widespread use of imaging has led to the increase in the incidence of small renal masses (renal tumours ≤4 cm) in the last decade, now representing 40%–50% of all new patients with RCC.^4^, ^5^ Partial nephrectomy (PN) has evolved as the standard treatment for cT1 tumours, although alternative nephron‐sparing strategies are also used for cT1a tumours, such as active surveillance (AS) and focal therapy (FT).^6^ For cT1 tumours, PN is the preferred treatment.^6^ However, when PN is considered risky in frail patients or when technically not feasible, radical nephrectomy (RN) is an alternative if the contralateral kidney has a normal renal function.^6^

A nationwide audit performed by the British Association of Urologic Surgeons (BAUS) between 2012 and 2016 demonstrated an association between annual hospital volume (HV) and the proportion of cT1 tumours treated with PN rather than RN (from 18.1% in centres performing <25 cases per year [lowest volume] to 61.8% in centres performing ≥100 cases per year [high volume]). This association persisted after adjustment for PADUA complexity. Furthermore, data from the BAUS audit revealed an inverse association between HV and complication rates for PN and RN.^7^

In the Netherlands, Aben et al. described trends in Dutch RCC care between 2010 and 2014.^8^ They found that most patients with RCC in the Netherlands were treated according to guidelines and observed a clear increase in PN over the years. In addition, variations between HV and hospital status (university hospitals, large general hospitals and community hospitals) became apparent.

Although post‐operative mortality is low, PN is recognised as a complex procedure with increased perioperative risk, such as bleeding, compared with RN.^9^, ^10^ In 2018, the Dutch Association of Urology (NVU) introduced the Dutch volume standard (DVS) in order to stimulate quality for hospitals performing PN and RN.^11^ According to the DVS, the minimum number of RNs is 10 per year, and for PNs, at least 10 procedures per year are required, calculated as mean over a period of 3 years.

Our objectives were to analyse clinical variation over time in the management of cT1 renal cancers in the Netherlands and to investigate the adherence to the DVS of hospitals performing surgeries for RCC.

## 2 MATERIALS AND METHODS

In this historic cohort study, all patients diagnosed with a cT1 RCC during 2014–2020 were identified in the Netherlands Cancer Registry (NCR), maintained by the Netherlands Comprehensive Cancer Organisation (IKNL). The NCR is a population‐based registry composed of data on all newly diagnosed cancer patients in the Netherlands and has nationwide coverage since 1989. The main source of notifications of new cancers is the automated nationwide network and registry of histo‐ and cytopathology (PALGA). In addition, cases of non‐pathology‐proven tumours are supplied to the NCR by the Dutch Hospital Data (DHD). After notification, independent and trained data managers routinely extract patient‐, tumour‐ and treatment‐related characteristics from medical records in all Dutch hospitals. Topography and morphology are coded according to the International Classification for Oncology (ICD‐O) third edition^12^ and disease stage according to the UICC Tumour‐Node‐Metastasis classification.^12^, ^13^

Treatment was categorised into five groups: RN, PN, FT, AS and others. It was assumed that patients with cT1 RCC without active treatment entered an AS protocol and were therefore classified as AS. Furthermore, PN, FT and AS were considered as nephron‐sparing strategies. To allow comparison, HV categories were based on the BAUS HV categories.^7^ It was not possible to use the exact BAUS HV categories because of smaller number of cases in the Netherlands. Therefore, HV categories were defined as follows: Hospitals performing surgeries were grouped according to their annual number of (partial) nephrectomies and categorised into low volume (1–25 nephrectomies per year), medium volume (25–49 per year) and high volume hospitals (>50 per year). Hospitals not performing surgeries for renal cancer were categorised as ‘hospital not performing surgeries’.

Hospitals performing surgeries were also categorised according to the 2018 DVS for both RN and PN. For total number of RN, hospitals were categorised into ‘not adhering to the DVS’ (one to nine RNs per year) and ‘adhering to the DVS’ (≥10 RNs per year). For PN, the threshold of the DVS is also set at 10 PNs per year but averaged over a 3‐year period. For each hospital, we calculated the average number of PNs over a 3‐year period. The calculated average number of PNs was assigned to the middle year of the 3‐year time period. For the last year of our study (2020), we calculated the average over the last 2 years (2019 and 2020). As some hospitals stopped performing (partial) nephrectomies during the study period, they were categorised to the ‘hospital not performing surgeries’ category from the moment they stopped performing this type of surgery. Those hospitals that performed one to three PNs per year were additionally checked by data managers for verification of this low number.

Descriptive analyses were performed to provide insight into patient characteristics. Trends in treatment over time were evaluated for cT1a and cT1b tumours separately.

Treatment variations were evaluated for cT1a and cT1b tumours diagnosed in 2019–2020 (after the introduction of the DVS) in both the DVS and HV categories and in hospitals not performing surgeries. Treatment patterns were also evaluated of referred and non‐referred patients separately, according to the DVS for total number of (partial) nephrectomies. Patients were categorised as referred when their hospital of diagnosis differed from the hospital where surgical treatment was performed. Furthermore, insight was obtained into the geographical distribution of cT1a tumours treated with FT. This was done by using the patient's zip code at the time of diagnosis in order to calculate the proportion of cT1a tumours treated with FT by province. The results by province were plotted in a geographical map for patients diagnosed between 2018 and 2020.

For surgically treated patients, descriptive analyses on patient and treatment characteristics were performed stratified by HV. Additional chi‐squared tests for categorical data and Mann–Whitney *U* test for continuous variables were used to evaluate differences. The percentage and trend of RNs and PNs performed in hospitals that comply with the DVS were calculated per year. The current volume standards were introduced in 2018. To evaluate trends in centralisation, earlier years were also included in these analyses as a reference.

All analyses were performed using Stata statistical software package (version 16.0). A *p*‐value of <0.05 was considered statistically significant. For this study, approval was obtained by the Privacy Review Board of the Netherlands Cancer Registry (K22.143).

## 3 RESULTS

In total, 10 964 patients with cT1 renal cancer were diagnosed in the Netherlands between 2014 and 2020, of which 59.7% were cT1a and 40.1% were cT1b. T‐substage of the cT1 tumour was unknown in 15 patients (0.2%). Median age at diagnosis was 68 years (IQR = 59–75). In total, 7120 (64.9%) patients were surgically treated, of which 53.8% were with PN and 46.2% were with RN.

### 3.1 Trends in treatment over time

**FIGURE 1:**
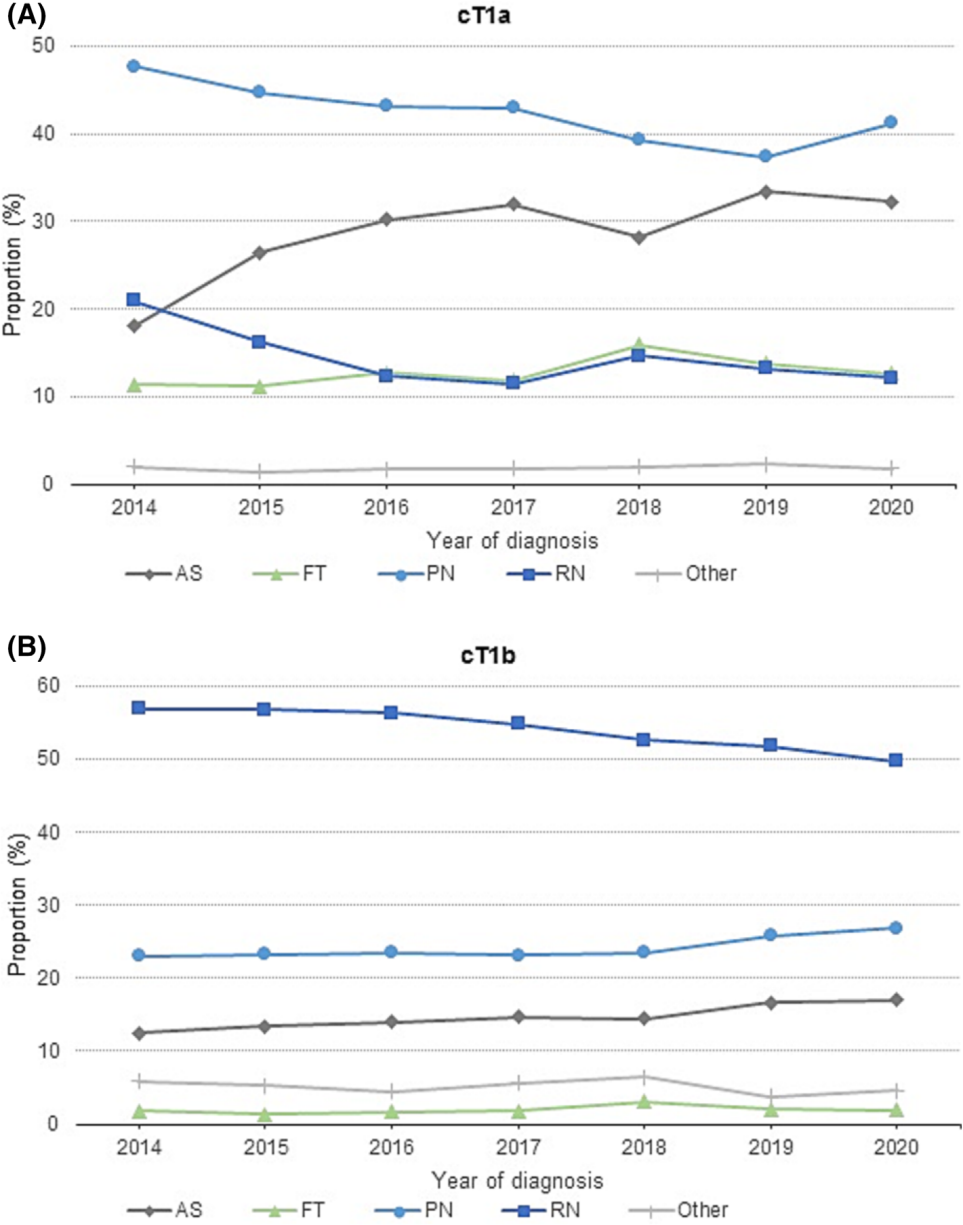
Distribution of treatment modalities over time (2014–2020). (A) For cT1a renal tumours. (B) For cT1b renal tumours. AS, active surveillance; FT, focal therapy; PN, partial nephrectomy; RN, radical nephrectomy.

From 2014 to 2020, the majority of patients with cT1a RCC were treated with PN, although over the years, the proportion of patients treated with PN decreased (from 47.7% in 2014 to 41.2% in 2020). An increase of AS was observed (from 18.0% in 2014 to 32.2% in 2020). Appliance of FT was stable during this study period for cT1a RCC and showed no change in 2020 compared with 2014 (±12%) (Figure 1A).

For cT1b tumours, RN was the most common treatment, although the overall proportion of RN decreased (from 56.8% in 2014 to 49.7% in 2020). A slightly increased use of PN was observed for cT1b, from 23.0% to 26.7%. AS was also applied more frequently over the years, from 12.5% to 17.0% (Figure 1B).

### 3.2 Treatment variations

**FIGURE 2:**
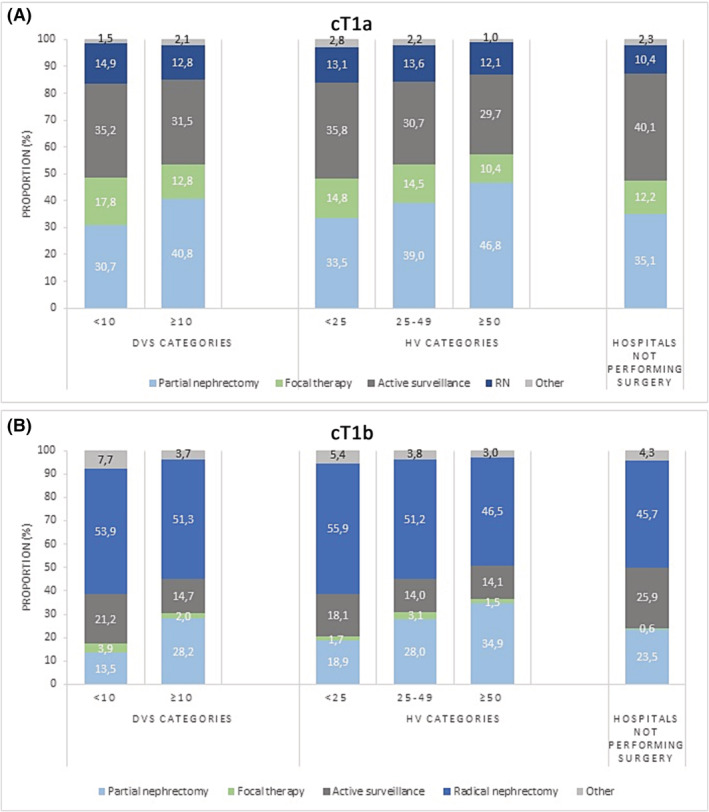
Treatment of patients diagnosed with renal cell carcinoma (RCC) in 2019 and 2020 in the Netherlands. Applied management is shown in different categories: 1. Patients diagnosed in hospitals that adhere and not adhere to the DVS; 2. Patients diagnosed in three hospital volume categories (<25, 25–49, >50 surgeries per year); 3. Patients diagnosed in hospitals not performing surgeries. (A) cT1a renal tumours. (B) cT1b renal tumours. DVS, Dutch volume standard; HV, hospital volume.

Variation in applied treatment was evaluated by HV. For cT1a tumours, approximately 85% were treated with nephron‐sparing management, independent of the HV category. However, differences in the applied nephron‐sparing strategy were observed between hospitals. Patients diagnosed in low‐volume hospitals and in hospitals not performing surgeries underwent AS more frequently. Fewer patients were treated with PN in low (33.5%) and medium (39.0%) volume hospitals compared with high‐volume hospitals (46.8%). FT was applied in 10.4%–14.8% of the patients and was mostly used in low‐volume hospitals. For cT1a, there was a percentage of patients treated with RN, approximately 12%–13% in all HV categories (Figure 2A).

For cT1b tumours, a higher proportion of patients diagnosed in high‐volume hospitals was treated with PN (34.9%) compared with medium (28.0%) and low HV (18.9%). As a consequence, fewer patients underwent a RN (46.5% vs. 51.2% vs. 55.9%, respectively). Patients diagnosed in hospitals not performing surgery (and referring patients to surgical hospitals) showed the lowest percentage of RN for cT1b (45.7%) but applied AS more often (25.9%) than PN (23.5%).

**FIGURE 3:**
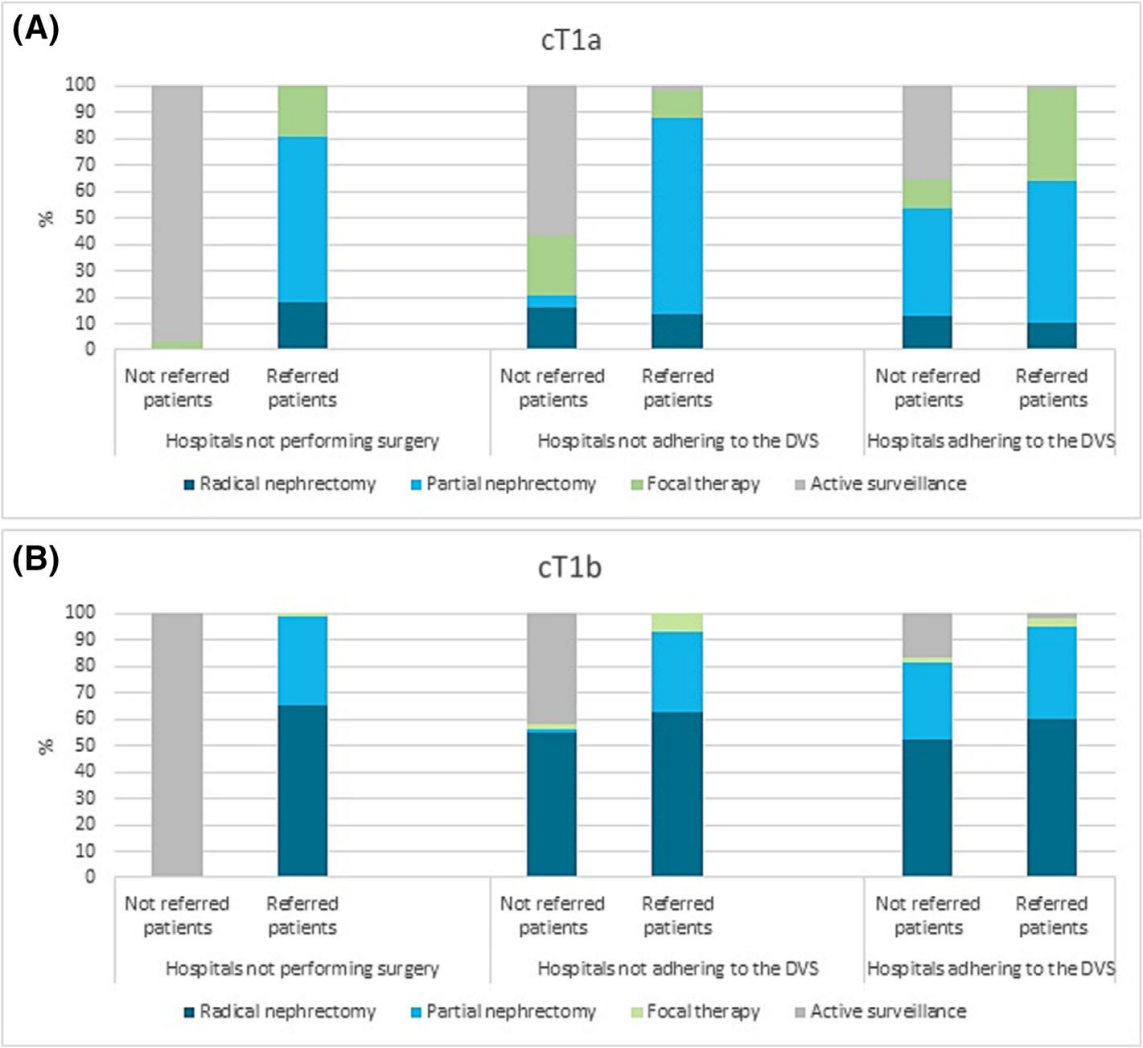
Referral patterns of patients diagnosed with renal cell carcinoma in 2019 and 2020 in hospitals not performing surgeries, hospitals not adhering to the DVS and hospital adhering to the DVS. (A) cT1a renal tumours. (B) cT1b renal tumours. DVS, Dutch volume standard.

**FIGURE 4:**
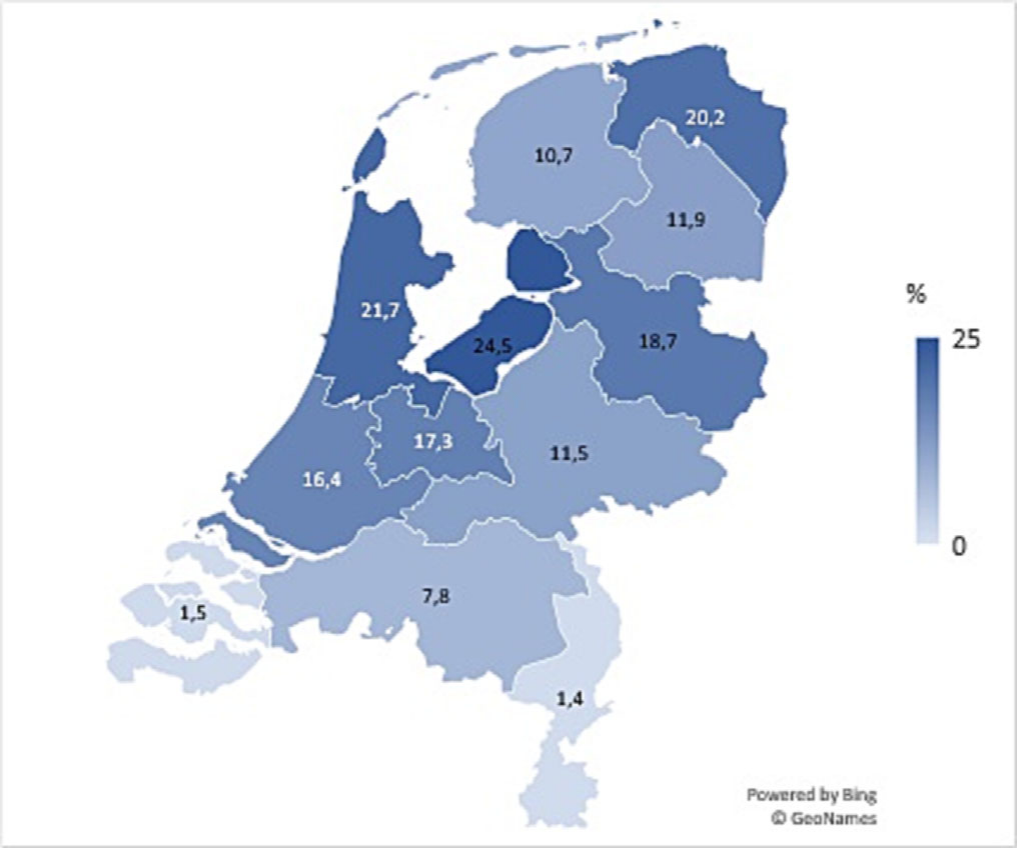
Geographical distribution of the proportion (%) of patients with cT1a renal cancer treated with focal therapy in the Netherlands in 2018–2020, based on the patients' ZIP code at the time of diagnosis.

Analysis of treatment patterns of referred and non‐referred patients revealed that hospitals not adhering to the DVS referred patients mainly for PN while managing FT, AS and some RN in their own hospital. FT is not included in the DVS but was evaluated in our study for referral patterns (Figure 3). In addition, geographical distribution showed large regional differences for patients that received FT for cT1a RCC, ranging from 1.4% to 24.5%, based on the zip code of the patient at the time of diagnosis (Figure 4).

### 3.3 Surgical treatment variation by HV

Surgically treated patients (PN or RN), diagnosed between 2014 and 2020, stratified by surgical HV (table 1), showed that almost half of the patients had their (partial) nephrectomy in a medium‐volume hospital. High‐volume hospitals treated relatively more cT1a tumours compared with medium and low‐volume hospitals.

**TABLE 1:** Characteristics of all surgically treated patients (*n* = 7120) diagnosed between 2014 and 2020 divided by surgical hospital volume category, and for cT1a and cT1b renal cell tumours.

Characteristic	Low HV <25 surgeries/year *N* (%)	Medium HV 25–49 surgeries/year *N* (%)	High HV >50 surgeries/year *N* (%)	*p*‐value
*N* cases	1470 (20.7)	3504 (49.2)	2146 (30.1)	‐
Gender				0.51*
Male	904 (61.5)	2210 (63.1)	1357 (63.2)	
Female	566 (38.5)	1294 (36.9)	789 (36.8)	
Median age at Diagnosis {IQR}	66 {58–73}	65 {56–72}	64 {55–71}	<0.01^**^
Clinical substage				<0.01*
cT1a	680 (46.2)	1795 (51.2)	1200 (55.9)	
cT1b	789 (53.7)	1706 (48.7)	942 (43.9)	
Unknown	1 (0.1)	3 (0.1)	4 (0.2)	
Type of surgery				<0.01*
PN	584 (39.7)	1845 (52.7)	1404 (65.4)	
RN	886 (60.3)	1659 (47.3)	742 (34.6)	
Surgical approach				<0.01*
Open	394 (26.8)	502 (14.3)	144 (6.7)	
Laparoscopic	889 (60.5)	2080 (59.4)	521 (24.3)	
Robot‐assisted	179 (12.2)	892 (25.4)	1437 (67.0)	
Unknown	8 (0.5)	30 (0.9)	44 (2.0)	
**T1a** (*n* = 3675)				
Type of surgery				<0.01*
PN	435 (65.0)	1333 (74.5)	996 (83.0)	
RN	245 (36.0)	462 (25.7)	204 (17.0)	
Surgical approach				<0.01*
Open	211 (31.0)	276 (15.4)	69 (5.7)	
Laparoscopic	364 (53.5)	930 (51.8)	201 (16.8)	
Robot‐assisted	99 (14.6)	574 (32.0)	905 (75.4)	
Unknown	6 (0.9)	15 (0.8)	25 (2.1)	
**T1b** (*n* = 3437)				
Type of surgery				<0.01*
PN	148 (18.8)	510 (29.9)	406 (43.1)	
RN	641 (81.2)	1196 (70.1)	536 (56.9)	
Surgical approach				<0.01*
Open	183 (23.2)	226 (13.3)	75 (8.0)	
Laparoscopic	524 (66.4)	1147 (67.2)	319 (33.9)	
Robot‐assisted	80 (10.1)	318 (18.6)	529 (56.1)	
Unknown	2 (0.3)	15 (0.9)	19 (2.0)	

Abbreviations: HV, hospital volume; IQR, interquartile range; PN, partial nephrectomy; RN, radical nephrectomy.

* Chi‐square test.

^**^ Mann–Whitney *U* test.

For cT1a, a higher proportion of patients was treated with PN in high vs. medium vs. low HV (83.0% vs. 74.5% vs. 65.0%, respectively, *p* < 0.01). For cT1b, 43.1% of the patients underwent a PN in high HV, compared with 29.9% in medium HV and 18.8% in low HV (*p* < 0.01).

Furthermore, HV seems to be related to the type of approach: Most (partial) nephrectomies in high‐volume hospitals were performed robot‐assisted (67.0%), whereas in medium‐ and low‐volume hospitals, the majority of (partial) nephrectomies were performed laparoscopically. In addition, low HV performed more surgeries with an open approach (27%), compared with 6.7% in high HV (*p* < 0.01; Table 1).

### 3.4 Adherence to the DVS

In total, 43 of 52 (82.7%) hospitals in 2020 did adhere to the DVS of at least 10 nephrectomies. For PN specifically, the number of hospitals performing PN adhering to the DVS was 26 of 42 in 2018 (61.9%), 25 of 42 in 2019 (59.5%) and 24 of 43 in 2020 (55.8%). Moreover, 19 hospitals in 2020 performed less than 10 PNs.

**FIGURE 5:**
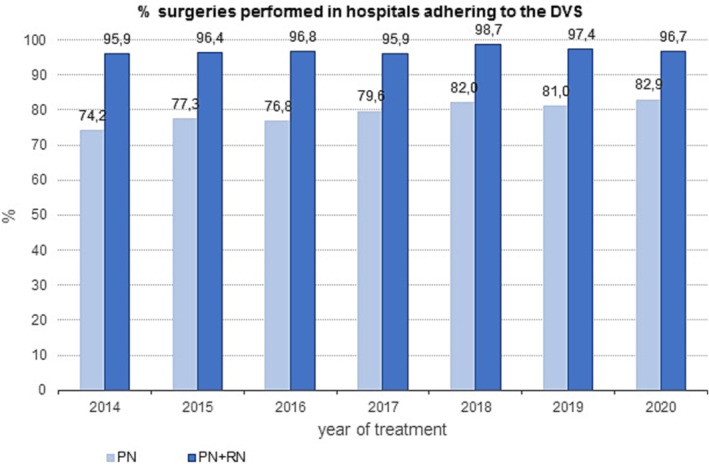
Proportion (%) of patients who underwent surgery that was performed in hospitals that adhere to the Dutch volume standard (DVS). PN, partial nephrectomy; RN, radical nephrectomy.

The total number of RN and PN performed in a hospital adhering to the DVS is shown in Figure 5. During the period 2014–2018, there was a trend of increasing proportion of PN performed in a hospital performing at least 10 PNs per year (74.2%–82.9%). After the introduction of the DVS in 2018, approximately 18% (17%–19%) of the PNs were performed in a hospital not adhering to the DVS (Figure 5).

## 4 DISCUSSION

This study shows that high‐volume hospitals showed closer guideline adherence compared with low and medium HV for T1 RCC. The EAU guidelines have recommended PN as preferred treatment for T1 RCC.^6^ The majority of patients with cT1a RCC were treated with nephron‐sparing options in all hospital categories, but variation in applied management was observed by HV, showing increasing numbers of patients treated with PN in high‐volume hospitals. For cT1b, there was an inverse correlation observed for RN and PN: The higher the HV, the lower the appliance of RN and more patients were treated with PN, suggesting closer guideline adherence. After the introduction of the DVS in 2018, still around 18% of PNs for cT1 tumours were performed in hospitals with an average of <10 PNs annually.

Our results are in line with a previous audit from the BAUS.^7^ In their study, Tran et al. analysed 13 045 surgically treated cT1 tumours between 2012 and 2016 in the United Kingdom. An association between HV and the proportion of cT1 tumours treated with PN rather than RN was found (also when subgrouping into cT1a and cT1b). This association persisted after adjustment for PADUA complexity. In the BAUS data, 18% of the cT1a tumours were treated with PN in hospitals with a volume of <25 surgeries per year, compared with 37% in hospitals with a volume of 25–49 surgeries per year, and 51% in hospitals with a volume of 50–99 surgeries per year. Hospitals with a volume of ≥100 surgeries per year showed that 62% of the patients with cT1a were treated by PN. Our observations are in line with the BAUS analysis in the United Kingdom, as our analysis showed fewer usage of PN in low‐volume hospitals compared with medium and high‐volume hospitals. Although the BAUS analysis was based on self‐reported data, our data were retrieved from a nationwide registration, collected by trained and independent data managers.

Another interesting finding of the BAUS audit was decreased complication rates with increasing HV for all patients, including patients treated with PN. PN is known to be a complex procedure and has been associated with higher complication rates compared with RN.^9^, ^14^ Other studies showed that undergoing robot‐assisted PN at higher volume hospitals has been associated with decreased risk of conversion, positive surgical margins and complication rates.^15^, ^16^ Arora et al. analysed outcomes after any PN in the United States in relationship with HV and attempted to identify an optimal HV threshold for performing PN. They found that decreased complication rates were associated with increasing annual HV, with plateauing seen at 35 to 40 PNs per year. In their study, robot‐assisted PN showed a similar association, with plateauing seen at 18 to 20 PNs annually.^17^ In our study, we could not analyse complication rates, as these data were not available. Future work should therefore be focused on a national registration of specialised care and surgery for RCC to improve clinical outcomes, decrease variations in practice patterns and subsequently increase guideline adherence in the Netherlands.

In an earlier study from the Netherlands, Aben et al.^8^ described guideline adherence for the management of cT1 RCC from 2010 to 2014. An increase in PN for cT1a tumours and a clear trend of decreasing usage of RN for cT1b tumours were observed over time. In addition, our study showed that the trend of decreased use of RN continued, although for cT1a tumours, the use of PN decreased over time, whereas a clear increasing trend of AS was found. Furthermore, Aben et al.^8^ found that treatment in a high‐volume hospital was associated with a higher probability of PN compared with RN for cT1a tumours. They hypothesised that referral from low‐ to high‐volume hospitals could partly explain the observed differences between low and high‐volume hospitals, although they did not analyse the referral patterns. Referral patterns in our study showed that hospitals not adhering to the DVS mostly referred patients for PN, while managing FT, AS and some RN themselves. This could partly explain the observed difference between low‐ and high‐volume hospitals in the usage of PN.

Another important observation in our study was the difference in surgical approach between different HV categories. Open nephrectomies were more common in low and medium HV compared with high HV. The majority of the (partial) nephrectomies in high‐volume hospitals were performed robot‐assisted. Laparoscopic RN is associated with less morbidity, shorter hospital stay and lower analgesic requirement compared with open RN.^18^ Robot‐assisted RN has not been proven superior over laparoscopic RN,^19^ although robot‐assisted PN is associated with lower conversion rates to open surgery, shorter warm ischemia time, smaller change in post‐operative GFR and shorter length of hospital stay compared with laparoscopic PN.^20^ Robot‐assisted PN has also shown superiority over open PN as well.^21^, ^22^ The question is however, if those surgeries performed open, could have been performed minimally invasive or if this was performed on specific indication. Without data of case‐mix such as nephrometry or comorbidity scores supporting this open approach for RN for cT1 tumours in low‐volume hospitals, we are unable to identify the rationale behind open (partial) nephrectomies.

FT showed a stable usage of around 12% for cT1a tumours in the Netherlands over time. Interestingly, based on the zip code of the patient at the time of diagnosis, we found that in certain regions, patients have better access to FT and that these data suggest less cross‐regional referral from regions in which FT is not available.

An explanation for differences in the type of nephron‐sparing management applied in the different Dutch regions could be inadequate use of shared decision‐making for cT1a RCC. T1 renal tumours are ideally suited for shared decision‐making, as several treatment options are available with their pros and cons and should be discussed with the patient.^23^, ^24^ It would be interesting in future studies to analyse the usage of shared decision‐making correlated to HV.

Some limitations should be addressed. Case‐mix data on tumour complexity (PADUA and/or RENAL score), patient comorbidity and complications were not available in the NCR, and therefore, these factors could not be taken into account in observed differences in treatment management between HV. Nevertheless, it is doubtful that low‐volume hospitals treated more complex tumours, as the BAUS data showed that in higher volume hospitals, more complex PNs were performed.^7^ Nevertheless, this would be very interesting to analyse in future studies, as this could perhaps explain the differences found in management variation between hospitals. In addition, despite that the registration was extracted by trained data managers, some (partial) nephrectomies might have been missed during the registration. The missing registrations could have resulted in hospitals that are just below the threshold, although a limited effect of possible missing registrations is expected.

Also, in our analysis, we did not include nephro‐ureterectomies (for urothelial cancer) and surgeries for benign lesions, such as oncocytoma, as the definition of the DVS does specifically mention oncological surgeries for RCC. Hospitals might have taken these surgeries into account in their adherence to the DVS.

Lastly, it should be mentioned that the impact of the COVID‐19 pandemic in 2020 was not evaluated in this study, which might have resulted in fewer PNs and could have prevented hospitals to reach the DVS threshold. The Dutch Association of Urology advised to delay surgery for cT1a low‐risk RCC during the COVID pandemic, and therefore, it is possible that PNs were postponed or treated otherwise.

Nevertheless, our study is based on a large nationwide registry with important information on differences in the management of cT1 RCC based on HV. We observed variation in applied management between different hospital categorisations. As fewer RNs were performed in high‐volume hospitals and PN was more often applied, it is questionable whether a volume standard with a minimum of 10 PNs per year is adequate, and therefore, an increase of the volume norms should be considered. With no data available on case‐mix of cT1 tumours in the Netherlands, a nationwide registry could be the solution to further understand the current differences in the management of cT1 RCC in the Netherlands.

## AUTHOR CONTRIBUTIONS

This paper was written by H. Yildirim. All analyses were performed by M. S. Schuurman. P. J. Zondervan, K. K. H. Aben, A. Bex and B. W. Lagerveld have made a substantial contribution to the research design and interpretation of data. All authors revised the paper and approved the final version.

## CONFLICT OF INTEREST

A. Bex: Recipient of a restricted educational grant from Pfizer for a neoadjuvant trial (remuneration made to the employer and sponsor of the trial). Steering committee member of adjuvant trials of BMS and Roche/Genentech. No other conflicts of interest to declare by the authors.
